# Group 5 Pulmonary Hypertension Resulting From Thyrotoxicosis During Pregnancy

**DOI:** 10.1016/j.jaccas.2025.106656

**Published:** 2026-04-01

**Authors:** Rosa Drummond, Lisa Forbess, Allison Lankford

**Affiliations:** a Department of Obstetrics, Gynecology, and Reproductive Sciences, University of Maryland Medical Center, Baltimore, Maryland, USA; b Department of Medicine, Heart and Vascular Center, University of Maryland Medical Center, Baltimore, Maryland, USA

**Keywords:** Graves’ disease, high-output heart failure, pregnancy, pulmonary hypertension, right heart catheterization

## Abstract

**Background:**

We present a case of uncontrolled hyperthyroidism during pregnancy associated with heart failure with preserved ejection fraction and pulmonary hypertension (pHTN).

**Case Summary:**

A 32-year-old woman at 22 weeks’ gestation was nonadherent to hyperthyroid medications. She presented with shortness of breath and was found to have pHTN on transthoracic echocardiography. Right heart catheterization revealed post-capillary pHTN, an elevated pulmonary artery occlusion pressure (33 mm Hg), and high cardiac output, consistent with group 5 pHTN. She was managed with antithyroid medications, corticosteroids, and aggressive diuresis, and she continued the pregnancy.

**Discussion:**

Pulmonary arterial hypertension is associated with right heart failure and is currently classified as modified World Health Organization class IV (pregnancy contraindicated). However, group 5 pHTN may be managed safely during pregnancy.

**Take-Home Message:**

This case highlights that group 5 pHTN has a different cardiac risk profile compared with group 1 pulmonary arterial hypertension, and right heart catheterization was instrumental in stratifying this risk and counseling the patient.

## Visual Summary

**Figure:**
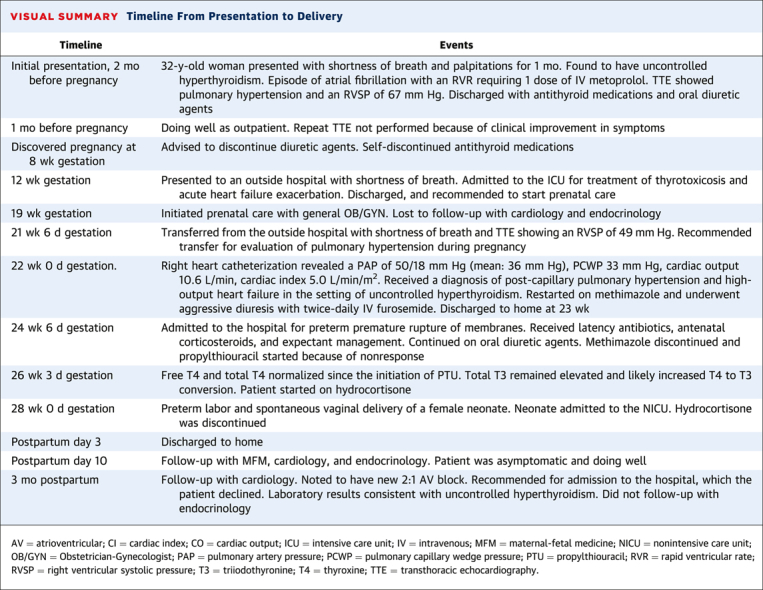

## History of Presentation

**Figure 1:**
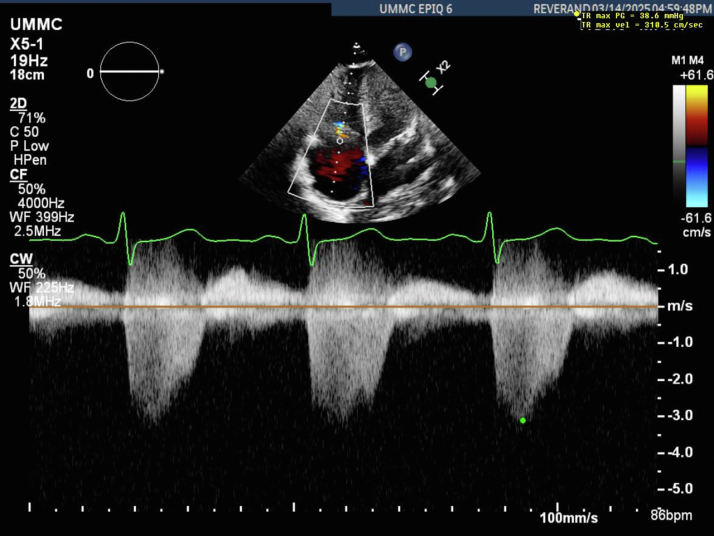
Transthoracic Echocardiography at 22 Weeks Gestation Patient presented at 22 weeks gestation with dyspnea on exertion. Tricuspid valve regurgitation with a TR max velocity of 320.0 cm/s, correlating with a right ventricular systolic pressure of 49 mm Hg. TR max velocity = tricuspid regurgitation maximum velocity.

A 32-year-old woman, gravida 4, para 1-0-2-1, at 22 weeks of gestation was transferred from an outside hospital with a month of worsening dyspnea on exertion in the setting of uncontrolled hyperthyroidism, high-output heart failure, and pulmonary hypertension (pHTN). Vital signs showed sinus tachycardia at 105 beats/min and hypertension, a respiratory rate of 40 breaths/min, and 100% peripheral oxygen saturation on room air. Physical examination revealed an enlarged, nontender goiter, clear auscultation of lung fields, regular cardiac rhythm, and 3+ pitting edema in the lower extremities. The fetal heart rate was normal. Hemoglobin was 7.7 g/dL, thyroid-stimulating hormone <0.01 mIU/L, free thyroxine (T4) 2.9 ng/dL, thyrotropin receptor antibody >40 IU/L, and N-terminal pro–B-type natriuretic peptide 2,410 pg/mL. Transthoracic echocardiography (TTE) revealed moderate dilation of all 4 chambers, left ventricular ejection fraction 60%, right ventricular systolic pressure (RVSP) 49 mm Hg, and tricuspid regurgitant jet velocity 3.2 m/s (Figure 1, Video 1).

## Past Medical History

**Figure 2:**
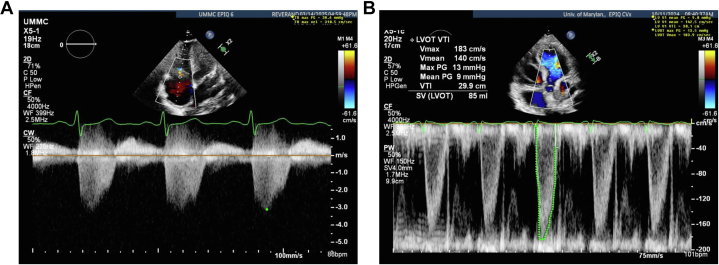
Transthoracic Echocardiography Prior to Pregnancy First presentation to the emergency department for dyspnea on exertion, immediately before pregnancy. (A) Tricuspid valve regurgitation with a TR max velocity of 385.0 cm/s, correlating with a right ventricular systolic pressure of 67.3 mm Hg. (B) The LVOT VTI is 29.5 cm, significantly elevated, consistent with volume overload. LVOT = left ventricular outflow tract; PG = pressure gradient; SV = stroke volume; TR max velocity = tricuspid regurgitation maximum velocity; Vmax = maximal velocity; Vmean = mean velocity; VTI = velocity time interval.

Two months before becoming pregnant, the patient presented to an outside hospital with 1 month of worsening shortness of breath and palpitations. Her past medical history included chronic hypertension and anemia. During that admission, she had an episode of atrial fibrillation with a rapid ventricular rate that resolved with 1 dose of intravenous metoprolol. The patient ultimately received a diagnosis of uncontrolled hyperthyroidism due to Graves’ disease. She was started on methimazole and propranolol. She had elevated N-terminal pro–B-type natriuretic peptide and peripheral edema, prompting TTE, which revealed a hyperdynamic left ventricle and an RVSP of 67 mm Hg, consistent with severe pHTN (Figure 2, Video 2). The patient was started on daily oral diuretic agents. Pulmonary function tests, computed tomography angiography for pulmonary embolism (PE), and laboratory work-up excluded parenchymal lung disease, PE, autoimmune disorders, and infectious causes of pHTN. After 4 days in the hospital, the patient was discharged with a plan for repeat TTE in 1 month and right heart catheterization (RHC) if pulmonary pressures remained elevated.

At 1 month after discharge, the patient did not undergo repeat TTE because of clinical improvement. She then became pregnant and was advised to discontinue oral diuretic agents and transition from methimazole to propylthiouracil (PTU), but she self-discontinued the medications due to pregnancy. At 12 weeks’ gestation, she presented to an outside hospital with shortness of breath and was admitted to the intensive care unit, where she was treated for thyrotoxicosis and acute heart failure exacerbation. The patient was discharged and recommended to establish prenatal care. She established prenatal care at 19 weeks’ gestation; however, she was lost to follow-up with endocrinology and cardiology.

## Differential Diagnosis

The differential diagnosis of new-onset pHTN is extensive, and management strategies are dependent on the underlying etiology. In a reproductive-aged individual, our differential primarily included pulmonary arterial hypertension (PAH), left-sided heart failure from cardiomyopathy or undiagnosed valvulopathies, autoimmune disorders, infection, or intraparenchymal lung disease. The patient had no history of venous thromboembolism to suggest chronic thromboembolic pHTN, and computed tomography angiography at the outside hospital was negative for PE. In addition, the patient denied a history of smoking or vaping. As PAH carries a uniquely elevated risk of perinatal morbidity and mortality during pregnancy, the patient was counseled on the necessity of identifying the etiology of elevated pulmonary pressures on TTE.

## Investigations

The patient underwent RHC at 22 weeks 0 days gestation, approximately 5 months after her initial diagnosis, which showed a pulmonary artery pressure of 50/18 mm Hg (mean: 36 mm Hg) and pulmonary capillary wedge pressure 33 mm Hg, consistent with post-capillary pHTN. Cardiac output and cardiac index were 10.6 L/min and 5.0 L/min/m^2^, respectively, consistent with high output even in the context of the elevated cardiac output of pregnancy. The patient did not have any procedural complications associated with RHC.

## Management

The patient received a diagnosis of World Health Organization group 5 pHTN due to high-output heart failure resulting from difficult-to-control hyperthyroidism and counseled on the importance of medication adherence to prevent worsening heart failure. Cardiology and endocrinology teams provided guidance on volume status and thyroid hormone control. The patient underwent aggressively diuresis with twice-daily intravenous furosemide. The patient's antithyroid medication regimen was changed from PTU to methimazole because of the risk of hepatotoxicity. Labetalol was substituted for propranolol to treat chronic hypertension. She was continued on daily oral furosemide. The patient was discharged from the hospital at 23 weeks with plans for close outpatient follow-up.

At 24 weeks 6 days, the patient reported leakage of fluid at a prenatal appointment and fetal tachycardia was noted. She received a diagnosis of preterm premature rupture of membranes and was admitted to the hospital for latency antibiotics, antenatal corticosteroids, and expectant management.

**Figure 3:**
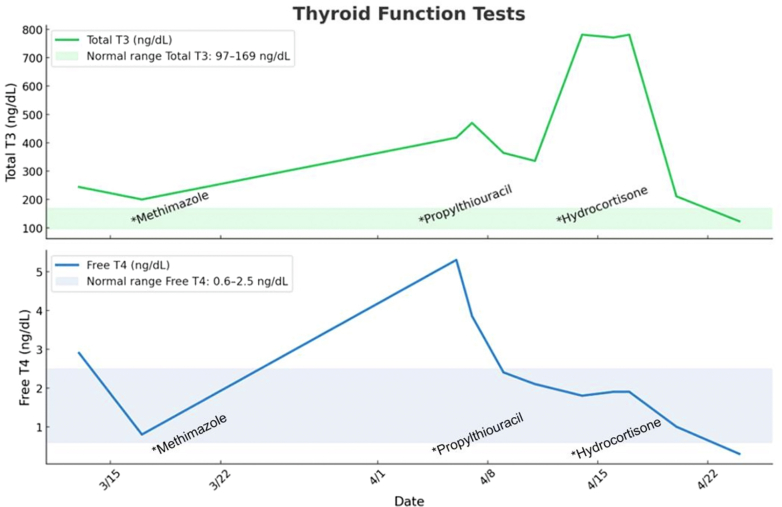
Thyroid Function Tests Over Time in Response to Antithyroid Medication and Hydrocortisone The patient did not respond to methimazole, and her thyroid function was uncontrolled. She did respond well to propylthiouracil. Hydrocortisone was added to reduce thyroxine to triiodothyronine conversion.

On admission, the patient was noted to be hyperthyroid with elevated free T4 (5.8 ng/dL; range: 0.6-2.5 ng/dL), total triiodothyronine (T3) (418 ng/dL; range: 97-169 ng/dL), and thyroid-stimulating immunoglobulin (>40 IU/L; range: <0.54 IU/L) despite adherence to her medications. There was no evidence of fetal goiter on ultrasound. The patient was not responding appropriately to methimazole; therefore, she was transitioned back to PTU. There was no evidence of hepatotoxicity. Although free T4 decreased to within the normal range, total T3 initially decreased but then quickly increased again. It was suspected that this was partly related to the antenatal corticosteroid effect wearing off, with the subsequent increased T4 to T3 conversion. Therefore, the patient was started on stress-dose corticosteroids with hydrocortisone 50 mg thrice daily, and ultimately thyroid hormones were controlled within the normal range (Figure 3). The patient was continued on daily oral furosemide, with a goal of a net negative volume status of 1 to 2 L daily.

## Outcome and Follow-Up

**Figure 4:**
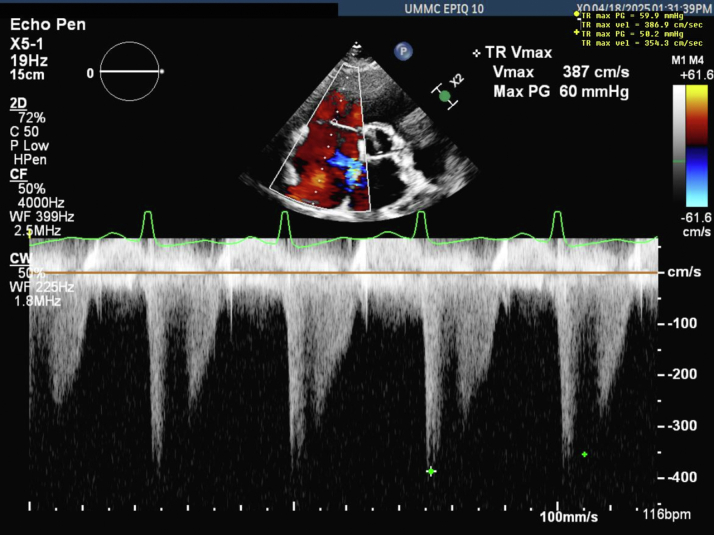
Transthoracic Echocardiography at 27 Weeks Gestation Patient was euthyroid at 27 weeks gestation, one week prior to delivery. Tricuspid valve regurgitation with a TR max velocity of 411.4 cm/s, correlating with an RVSP of 67 mm Hg. There is an increase in estimated RVSP because of physiological changes during pregnancy despite improvement in hyperthyroidism. RVSP = right ventricular systolic pressure; other abbreviations as in Figure 2.

The patient was monitored closely in the hospital. She was continued on PTU, labetalol, and hydrocortisone. At 27 weeks, the patient was noted to be euthyroid, and repeat TTE showed normal right ventricular size and function, with no evidence of right ventricular strain or flattening of the interventricular septum (Figure 4, Video 3). At 28 weeks’ gestation, hydrocortisone was discontinued when thyroid function tests showed low free T4 and total T3 levels. However, the next day she experienced preterm labor and had a spontaneous vaginal delivery of a female neonate. The patient tolerated labor and delivery well from a cardiovascular perspective without any adverse cardiac events and was discharged on postpartum day 3. The neonate was admitted to the neonatal intensive care unit due to prematurity and was treated for neonatal hyperthyroidism with methimazole. The neonate otherwise had a routine course in the neonatal intensive care unit and was discharged to home at 37 weeks’ corrected gestational age.

## Discussion

*pHTN* is defined by a mean pulmonary artery pressure >20 mm Hg measured by RHC.^1^^,^^2^ It is a heterogeneous disease process with multiple etiologies. Under normal pregnancy physiology, there is an increase in cardiac output by 30% to 50% and thus increased flow within the pulmonary vasculature. In a healthy circuit, pulmonary arterial pressure remains unchanged because of the normal hemodynamic and hormonal adaptations of pregnancy, including a decrease in systemic and pulmonary vascular resistance.^3^ However, in patients with PAH, there is decreased compliance in the pulmonary vascular system and subsequent inability to tolerate increased cardiac output. This leads to an increase in right ventricular afterload, right ventricular dysfunction, and ultimately right heart failure, leading to cardiogenic shock.^4^ For this reason, primary PAH (group 1) is a contraindication to pregnancy due to the high risks of perinatal morbidity and mortality, with historic mortality rates as high as 11% to 56%.^5^, ^6^, ^7^

TTE with color Doppler velocimetry of regurgitant flow across the tricuspid valve is a noninvasive method to investigate right-sided cardiac pressures. Although TTE has many useful applications, Fisher et al^8^ found that Doppler measurements can over- or underestimate pulmonary artery systolic pressure by 10 mm Hg in up to 48% of nonpregnant patients. In contrast, Herrera et al^9^ found that mean pulmonary artery pressure was overestimated in pregnant patients compared with measurements obtained via RHC. Although TTE may be useful to identify a patient with elevated pulmonary artery systolic pressure, it is often unable to differentiate between pre-capillary and post-capillary pHTN. Cardiac catheterization during pregnancy carries the risks of fetal radiation (highest risk in the first trimester), thrombosis, bleeding, and arrhythmia, and therefore it is not undertaken routinely. However, in our patient with an elevated RVSP at 22 weeks’ gestation, it was imperative to diagnose the etiology of pHTN to counsel her appropriately regarding her risks in continuing the pregnancy. Once the patient received the diagnosis of group 5 pHTN, treatment of her high-output heart failure could be addressed, and the morbidity and mortality risks could be attenuated to those of left ventricular diastolic dysfunction associated with volume overload.

Because of the patient's history of nonadherence to her antithyroid medications and laboratory evidence of severe hyperthyroidism, her clinical presentation with shortness of breath and peripheral edema was attributed to high-output heart failure secondary to uncontrolled hyperthyroidism. Graves’ disease during pregnancy can be well managed with antithyroid medications, but poorly controlled disease can lead to maternal, fetal, and neonatal complications including thyroid storm, heart failure and cardiogenic shock, preeclampsia, fetal growth restriction, fetal goiter and hyperthyroidism, preterm birth, and stillbirth.^10^ Most patients can be managed with antithyroid medications, including PTU in the first trimester and methimazole in the second and third trimesters. PTU is usually discontinued after the first trimester because of the risk of hepatotoxicity. In this unique case, the patient did not respond to methimazole and was transitioned back to PTU at 24 weeks’ gestation when she was readmitted to the hospital. However, our patient continued to have a high rate of T4 to T3 conversion refractory to high doses of PTU. With the addition of 10 days of high-dose corticosteroids, euthyroidism was achieved.

The patient's group 5 pHTN was successfully managed during pregnancy by treating her hyperthyroidism and high-output heart failure with medical management.

## Conclusions

PAH is associated with right heart failure and is classified as modified World Health Organization class IV (pregnancy contraindicated). Although group 5 pHTN is not classified under modified World Health Organization, patients remain at risk for adverse cardiac events and, to manage the pregnancy safely, management by a dedicated cardio-obstetrics team is warranted. Arriving at the correct diagnosis via RHC was critical in the management of this patient.

## Funding Support and Author Disclosures

The authors have reported that they have no relationships relevant to the contents of this paper to disclose.

**Take-Home Messages**

• Group 5 pulmonary hypertension has a different cardiac risk profile compared with group 1 pulmonary arterial hypertension.
• Right heart catheterization was instrumental in stratifying this risk and counseling the patient.

## Appendix

Video 1 Patient Presented at 22 Weeks’ Gestation With Dyspnea on Exertion The apical 4-chamber view shows a mildly dilated left ventricle with preserved systolic function. The parasternal long-axis view shows a mildly dilated right ventricle with preserved systolic function. The estimated RVSP is 49 mm Hg.

Video 2 First Presentation to the ED for Dyspnea on Exertion, Immediately Before Pregnancy The parasternal short-axis view shows RV strain with flattening of the interventricular septum, and the apical 4CH view shows moderate biatrial enlargement.

Video 3 Patient Euthyroid at 27 Weeks’ Gestation, 1 Week Before Delivery The parasternal short-axis view shows normal RV size and function. There is no longer with evidence of RV strain or flattening of the interventricular septum. The left ventricle is normal in size. There is normal left ventricular wall thickness. The ejection fraction is 65% to 70%. Left ventricular systolic function is normal. The left ventricular wall motion is normal.
